# Effect of Using Oleogel on the Physicochemical Properties, Sensory Characteristics, and Fatty Acid Composition of Meat Patties

**DOI:** 10.3390/foods13233849

**Published:** 2024-11-28

**Authors:** Aidyn Igenbayev, Mukhtarbek Kakimov, Maigul Mursalykova, Bartosz Wieczorek, Bożena Gajdzik, Radosław Wolniak, Damian Dzienniak, Michał Bembenek

**Affiliations:** 1 Department of Food Technology and Processing Products, S. Seifullin Kazakh Agrotechnical Research University, Zhenis Avenue 62, Astana 010011, Kazakhstan; aidyn_mamyt@mail.ru (A.I.); maigul_85@mail.ru (M.M.); 2 Institute of Machine Design, Faculty of Mechanical Engineering, Poznan University of Technology, Piotrowo 3, 60-965 Poznan, Poland; bartosz.wieczorek@put.poznan.pl; 3 Department of Industrial Informatics, Faculty of Materials Engineering, Silesian University of Technology, 44-100 Gliwice, Poland; 4 Department of Economics and Informatics, Faculty of Organization and Management, Silesian University of Technology, 44-100 Gliwice, Poland; rwolniak@polsl.pl; 5 Department of Manufacturing Systems, Faculty of Mechanical Engineering and Robotics, AGH University of Krakow, A. Mickiewicza 30, 30-059 Krakow, Poland; ddamian@agh.edu.pl (D.D.); bembenek@agh.edu.pl (M.B.)

**Keywords:** meat patties, oleogel, PUFA composition, physicochemical parameters, sensory characteristics, organoleptic characteristics, structural characteristics

## Abstract

This study investigated the physicochemical properties of meat patties, comparing a control sample and an experimental sample with the addition of 10% oleogel. The experimental sample showed a reduction in protein content (19.47%) and fat (18.37%) compared with the control sample (20.47% and 19.95%, respectively), accompanied by an increase in carbohydrates (2.56% vs. 1.65%). The fatty acid composition analysis revealed that the inclusion of oleogel significantly increased the content of polyunsaturated fatty acids (PUFAs) from 12.458% to 18.94%. Saturated fatty acids (SFAs), such as capric, lauric, myristic, and stearic acids, were markedly reduced, while the level of linoleic acid increased, indicating an improved and balanced fatty acid profile in the experimental patties. The moisture-binding capacity of the experimental sample was determined to be 75.54%, a 2.53% improvement over the control one. Microstructural analysis found no significant differences between the control and experimental samples, with no large oleogel particles visible. Overall, the substitution of pork fat with oleogel did not adversely affect key physicochemical properties, such as pH and moisture-binding capacity, or the structural integrity of the beef patties. These findings suggest that oleogel can be used effectively as a fat substitute in meat products, enhancing their nutritional profile without compromising quality.

## 1. Introduction

The replacement of animal fats in meat products with plant-based fats is currently undergoing significant development. There has been a growing focus on substituting animal fats with monounsaturated fatty acids (MUFAs) and polyunsaturated fatty acids (PUFAs) without compromising the taste of the final meat products [1,2].

The semi-finished meat industry is also experiencing substantial growth, driven by the global popularity of these products. The rising demand from health-conscious consumers for safer, high-quality meat products poses a new challenge to researchers and producers in the meat industry: the need to develop low-fat, healthier alternatives [3,4].

The increase in the number of chronic diseases is closely related to the increased content of saturated and trans fatty acids in foods, especially in meat products. Moreover, high levels of saturated fatty acids (SFAs) and cholesterol cause serious health problems [5,6,7,8].

In response to this, numerous health organizations and government agencies have implemented measures aimed at reducing saturated fats in food, spurring the development of low-fat products and alternative fatty acid formulations within the food industry [9].

One solution to this challenge is the creation of structured oleogels. Structuring healthy fats into oleogels offers a way to produce functional ingredients with solid properties similar to animal fats, making them suitable for use in meat product manufacturing [10]. Edible oleogels, which can form liquid fats, are a promising alternative to traditional oils. Current research on oleogels focuses on the creation of unsaturated fats with varying fatty acid contents, but owing to the high level of unsaturation of the fats used, lipid oxidation remains a significant problem in maintaining product quality [11]. Food oleogels have emerged in response to the need to replace animal fats with vegetable oils, along with the use of structured organogel additives. Monoglycerides, natural wax, phytosterols, and ethyl cellulose are some of the common structuring agents used in meat products, such as sausages, patties, and liver pâtés [12,13,14].

Oleogels, which are added to food products, have become a major innovation in food production, primarily because of their ability to enhance the nutritional properties and structural characteristics of food by incorporating healthy fats [15,16,17]. For example, canola oil has been successfully structured using hydroxypropyl methylcellulose (HPMC) to create solid oleogels, with studies evaluating the potential HPMC oleogels as substitutes for animal fats (such as beef fat) to reduce saturated fat levels in meat pies [18].

Researchers have also explored using gel-like emulsion systems (GE) containing peanut and linseed oils to partially or completely replace beef fat in emulsified sausages. This resulted in a 40% reduction in total fat content and a 27% decrease in calorie count. Additionally, SFAs and cholesterol in these sausages were successfully reduced, while the content of MUFAs increased significantly [19]. These semi-solid systems, developed through the rational use of waxes and fatty acid derivatives as oleogels, present new possibilities for regulating the properties of oleogels for both food and functional applications [20].

In a study by Badar I. H. et al., the replacement of animal fat in burgers with vegetable oil successfully reduced SFAs while increasing PUFAs, particularly linolenic acid [21]. The present study aims to investigate the effects of replacing pork fat with 10% oleogel on the physicochemical properties, fatty acid composition, and structural characteristics of meat patties.

## 2. Materials and Methods

### 2.1. Materials

The meat used for the patties was sourced from the “ARQA ET” farm in Usambai village, Yerementau district, Akmola region. The cattle were pasture-fed on natural grass. For the experiment, first-grade beef from the femoral part of the Red Steppe breed was used, along with lean pork from the corresponding region of the thigh.Beeswax, employed as an oleogel stabilizer, was obtained from the Katon-Karagai district in eastern Kazakhstan. The key characteristics of the beeswax are as follows: pale yellow color, the distinctive smell of natural beeswax, homogeneous and pasty consistency, and a melting point of 72 °C.

Sunflower oil meeting the technical specifications outlined in Kazakh ST RK GOST R 52465-2010 was used, ensuring compliance with food industry standards [22].

The monoglyceride, with a minimum content of 95%, was supplied by Kerry Ingredients (M) Sdn Bhd, Malaysia. It has an acid value of no more than 3 mg KOH/g and an iodine value not exceeding 3 g I_2_/100 g.

### 2.2. Technology of Oleogel Production

**Figure 1:**
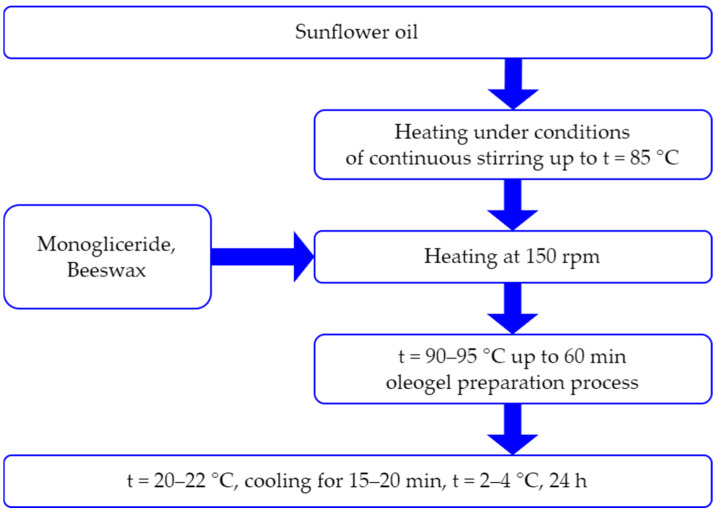
Technological diagram illustrating the process of obtaining oleogel.

The technology for producing oleogel, which was incorporated into meat patties, is illustrated in Figure 1. Oleogels were prepared following a modified procedure based on previous studies [23].

The process of obtaining oleogel and the ingredients used in oleogel production are depicted in Figure 1.

**Figure 2:**
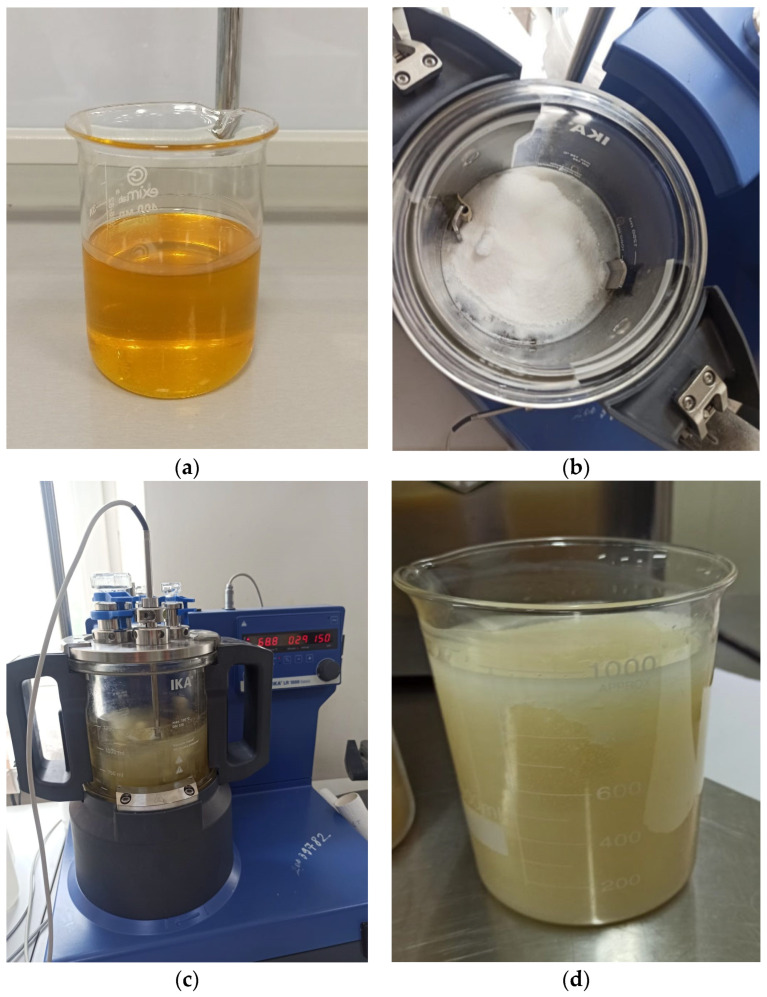
Oleogel production process: (**a**) beeswax, (**b**) monoglyceride, (**c**) obtaining oleogel, (**d**) obtained oleogel.

The process was conducted in the LR 1000 laboratory chemical reactor (IKA Werke GmbH & Co., Breisgau, Germany), equipped with an upper stirrer, at a temperature of 90–95 °C for 60 min, with continuous stirring at a speed of 150 rpm. The oleogels were then cooled and stored at 4 °C for 24 h to stabilize their desired structure. The entire process of producing oleogel is shown in Figure 2.

### 2.3. Preparation of Meat Patties

Two formulations were prepared: a control sample and an experimental sample. The control sample consisted of 60% beef, 30% pork, and 10% pork fat, along with salt and spices (Table 1). In the experimental sample, the 10% pork fat was substituted with an equal amount of oleogel while maintaining the same proportions of the other ingredients.

**Figure 3:**
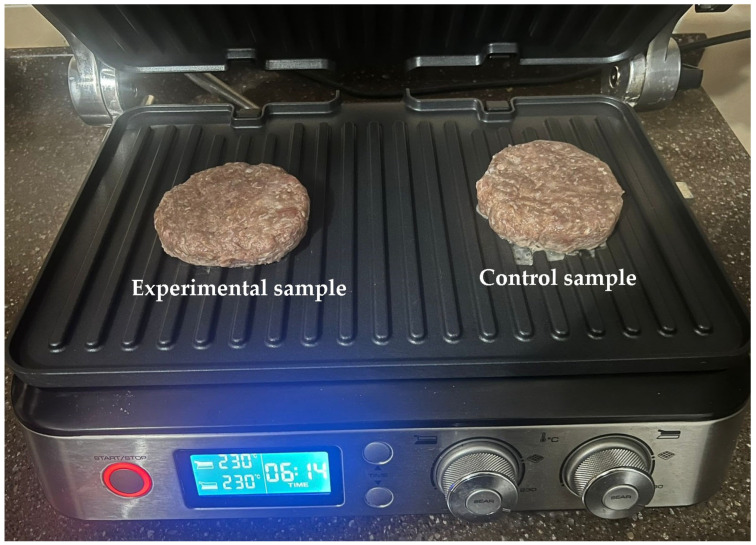
Heat treatment of the meat patty samples.

Beef, pork, and pork fat were ground using the MIM-300 meat grinder (Torgmash, Russia) with a hole diameter of 2–4 mm. The main ingredients were mixed for 11 min along with the additional ingredients. The mixture was then formed into 70 g patties using the ELF 4M IPCS-123 (N) automatic patty machine (Russia), yielding a total of 60 meat patties (30 control and 30 experimental samples). The patties were frozen at a temperature of −18 ± 2 °C and stored at −6 ± 1 °C. Figure 3 shows the appearance of the prepared meat patties. Prior to testing, they were cooked in the De’Longhi MultiGrill CGH1012D contact grill (China) at 230 °C for 6 min simultaneously on both sides.

### 2.4. Determination of Chemical Composition

The chemical composition of meat from cattle at various ages—specifically 16 to 18 months, 36 to 48 months, and 72 to 96 months—was investigated. The study involved analyzing 12 to 15 samples for each age group, and the average values obtained are presented in Table 1.

The content of moisture, protein, ash, and fat was determined in accordance with ISO standards [24,25,26].

- Moisture Content: In order to assess moisture content, the samples were first dried in furnaces at 105 °C until a constant weight was achieved. The moisture loss was then calculated.
- Protein Content: The protein content was determined using the Kjeldahl method, which involves the mineralization of organic substances in the sample followed by measuring the nitrogen content based on the amount of ammonia produced [27].
- Total Ash Content: The total ash content was measured by incinerating the sample at a temperature of 550 ± 25 °C. The mass of the residue, which comprises minerals resulting from salinization, was subsequently calculated.
- Fat Content: The fat content was derived by subtracting moisture, fat, protein, and ash from a total of 100 g of the sample.

All analyses were conducted with a minimum of 15 replicates to ensure accuracy and reliability.

### 2.5. Determination of pH

Acidity was measured using a potentiometric method with a pH-tester 340 (Infraspak-Analit, Novosibirsk, Russia). Prior to analysis, the pH-tester was calibrated with a standard solution mixed with distilled water. Calibration was performed by immersing the two electrodes of the pH-tester into the solution and taking readings. To prepare the sample solution, meat patty samples were finely ground and mixed with distilled-deionized water at a ratio of 1 part meat to 10 parts water. The mixture was allowed to infuse for 30 min at 20 °C, after which the pH readings were taken [28].

### 2.6. Determination of Moisture-Binding Capacity

The control and experimental samples (containing 10% oleogel) were mixed in a meat grinder for 5, 7, 9, 11, and 13 min to assess for the moisture-binding capacity of the sliced meat patties. To evaluate the moisture-binding properties, 3 g of sliced meat was taken from each sample, placed on filter paper, and pressed under a 1 kg weight for 10 min. The resulting meat moisture spots on the filter paper were scanned using the KOMPAS-3D v14 program, and the obtained images were saved in JPEG format. Next, the image file was opened in KOMPAS-3D v14, and the “AREA” command was used to show the boundaries of the inner adjacent areas corresponding to the moisture spots. The software then calculated the total average value of these adjacent areas, providing a quantitative measure of the moisture-binding capacity [29].

### 2.7. Determination of Yield Stress of Minced Meat Batter

The yield stress of the minced meat batter was determined using the ST-2 structurometer (Russia). The analysis of the results was performed automatically on a computer, utilizing the “Strukturometr ST-2” software designed specifically for the ST-2 structurometer. Prior to the experiment, the device’s operating mode was configured according to the methodological guidelines for tool selection, equipment, and sample preparation.

For measuring the yield stress of the minced meat sample, a cone indenter with a 60° cone angle was employed. The sample was carefully packed with a spatula into a cylindrical container. This container was positioned on the table in perfect alignment with the instrument’s axis.

**Figure 4:**
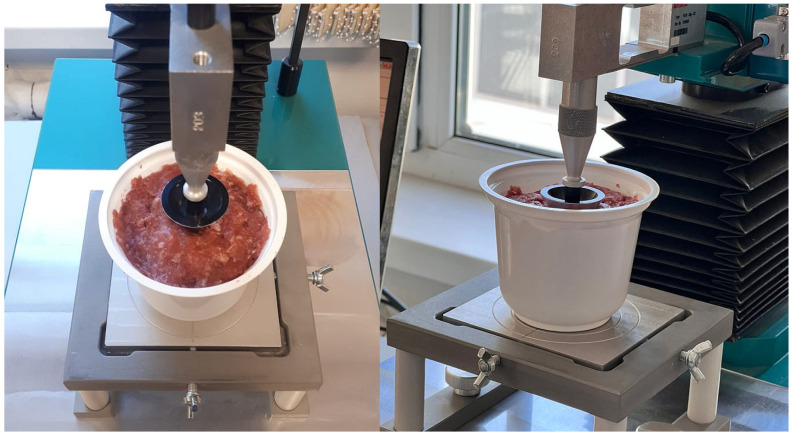
The process of conducting the test using the ST-2 structurometer.

Next, the indenter was positioned at the optimal location for measurement using the dedicated software. Once the position was set, the “start” button was pressed to initiate the automatic movement of the cone indenter, which was then immersed into the sample, as shown in Figure 4.

The software automatically captures the instrument readings. After the cone has been immersed and the instrument reactivated, the procedure is finalized by pressing the “stop” button. The experimental data are then saved in the form of tables and graphs.

Conical plastometers, as developed by P. A. Rebinder and N. A. Semenenko, are widely utilized in rheological studies due to their simplicity and reliability.

The yield stress of the minced meat was determined based on the depth of immersion of the cone (Equation (1)):

(1) $$\psi =K\frac{F}{h^{2}}$$

where F is applied load (N); h is the total penetration depth of the cone (m); and K is the constant of the cone, which depends on the cone’s vertex angle α (—).

Since the device provides the load in grams and the cone depth in millimeters, for convenience in calculations, grams must be converted to newtons. The result is expressed in pascals (Pa).

The constant K, which corresponds to the cone angle α at the top, is calculated using the formula shown in Equation (2):

(2) $$K=\frac{\cos^{2}\left(\frac{\alpha }{2}\right)}{\pi \tan\left(\frac{\alpha }{2}\right)}$$

where α is the cone’s vertex angle.

In this case, the vertex angle of the cone was 60°.

### 2.8. Determination of Fatty Acid Composition

The method used to determine the fatty acid content allows for the extraction of 90–95% of all cellular lipids. Lipid extraction was performed using a mixture of hexane and heptane in a 2:1 ratio. For each sample, 50 g of it was homogenized by grinding in a porcelain mortar with a pestle.

The homogenized sample was then transferred to a 500 mL flask, to which 200 mL of hexane and 100 mL of heptane were added as solvents to precipitate proteins and extract fatty acids. The flask containing the sample and solvents was placed on a shaker and mixed thoroughly at 180 rpm for 3–4 h, allowing for effective extraction. The resulting liquid was evaporated in a water bath at 70 °C.

The concentrated solution underwent acid-catalyzed esterification to obtain the corresponding fatty acid methyl esters (FAMEs). After evaporation, the sample was transferred to an Eppendorf tube and placed in a freezer for further analysis.

The fatty acid content in the meat patties was determined using gas chromatography on the Agilent 6890N gas chromatograph (Agilent Technologies, Santa Clara, CA, USA), equipped with a flame ionization detector. A CP-Sil 88 column (Chrompack, Middelburg, The Netherlands) was used, with dimensions of 100 m in length, 0.25 mm in diameter, and a film thickness of 0.2 μm. The analysis was conducted in accordance with GOST 31754-2012 [30], at a column temperature of 175 °C and a carrier gas linear velocity of 19 cm/s.

### 2.9. Evaluation of Sensory Characteristics of Meat Patties

Sensory analysis was conducted by 20 trained experts (13 female and 7 male) from Saken Seyfullin Kazakh Agrotechnical University. Testing took place in a room equipped with individual tasting booths under white light, following ISO procedures [31]. During the sensory evaluation of the meat patties, jury members assessed various parameters, such as smell, taste, texture, and appearance, using a five-point scale ranging from 1 (very disliked) to 5 (very liked). This evaluation allowed the experts to provide a comprehensive assessment of the quality and acceptability of the meat patties. Sensory sessions comprised seven repetitions, and average values for the indicators were obtained.

### 2.10. Microstructure Analysis of Meat Patties

The microstructure of the meat patty samples was examined under the JSM-6390LV JEOL low-vacuum scanning electron microscope (Tokyo, Japan).

### 2.11. Statistical Analysis

The experiments were conducted in five repetitions to ensure accuracy, and standard deviation values were calculated for all measurements. Differences between the experimental and control samples were assessed using a one-way ANOVA, followed by a Tukey post-hoc test. Statistical significance was determined at a threshold of *p* ˂ 0.05. For the sensory tests, a non-parametric Mann–Whitney *U* test was employed.

## 3. Results and Discussion

### 3.1. Physicochemical Characteristics of Meat Patties

This study investigated the effects of replacing pork fat with oleogel in meat patties. The physicochemical parameters of the meat patties of the control and experimental samples are shown in Table 2.

The replacement of pork fat with oleogel had a significant impact on the fat content, reducing it by approximately 7.9%. This reduction aligns with the initial goal of developing a healthier product with lower fat content while maintaining the textural and sensory characteristics of the patties. The slight increase in moisture content in the experimental sample (+1.2%) suggests that the oleogel may have enhanced moisture retention. No significant changes were observed in the protein, carbohydrate, or ash content.

These results can be attributed to the fact that the ingredient composition across all the first-grade beef patty formulations remained identical, except for the replacement of 10% pork fat with oleogel in the experimental sample. This substitution resulted in a minor decrease in both fat and moisture content. The physicochemical characteristics of the first-grade beef patties proved to be consistent with previous studies on meat patties [32,33,34,35].

In summary, the experimental sample with oleogel exhibited a slightly reduced protein content and increased moisture, carbohydrate, and pH values compared with the control sample. These findings suggest that the oleogel may have influenced the chemical composition and properties of the meat patties, potentially affecting their texture, flavor, and overall quality.

### 3.2. Moisture-Binding Capacity of Meat Patties

**Figure 5:**
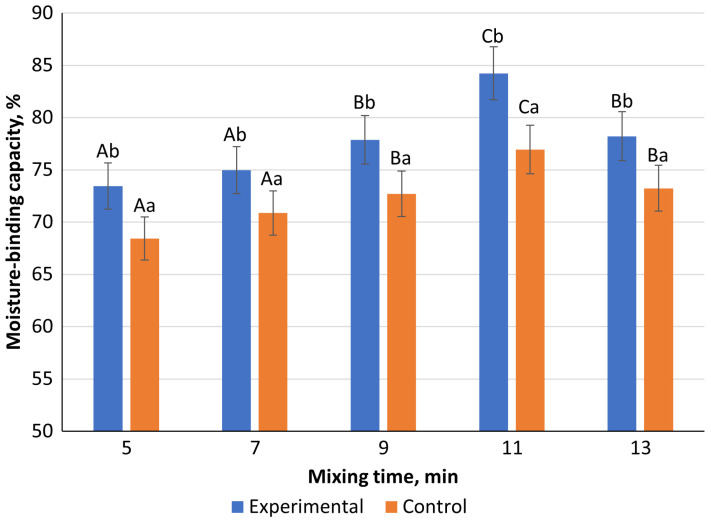
Moisture-binding capacity of meat patties. Note: Different lowercase letters (a–b) within the same column indicate statistically significant differences between the control and experimental samples (*p* < 0.05). Different uppercase letters (A–C) indicate a significant difference within the same type of sample. The bars have the form mean value ± standard error.

The results of the moisture-binding capacity of control and experimental meat patty samples are presented in Figure 5. Control and experimental samples (with 10% oleogel) were mixed in a minced meat mixer at a speed of 150 rpm for periods of 5, 7, 9, 11, and 13 min, and the moisture-binding capacity of first-grade beef patties was investigated.

The results of the study (Figure 5) showed that the moisture-binding capacity varied between the control and experimental samples across the mixing times. The highest moisture-binding capacity was observed after 11 min of mixing, with the control sample reaching 73.01 ± 0.91% and the experimental sample (containing oleogel) achieving 75.54 ± 0.86%, a noticeable increase of 2.53%. This suggests that the inclusion of a fat emulsion, such as oleogel, improves the moisture-binding capacity of meat patties, as indicated also in previous studies [36,37].

### 3.3. Results of Yield Stress of Minced Meat Batter

The effect of oleogel substitution on the rheological properties of the meat batter was investigated by measuring yield stress during the mixing process. Yield stress measurements were taken after 5, 7, 9, 11, and 13 min of mixing for both sample types (Table 3). Statistical analysis revealed no significant differences between the control and experimental samples at any mixing time point, indicating that the substitution of pork fat with oleogel did not affect the yield stress of the meat patties during mixing. This suggests that oleogel can serve as a viable alternative to pork fat without altering the rheological properties of the meat matrix during processing [38].

Within both sample groups, significant differences (*p* < 0.05) were observed when comparing yield stress values at specific mixing times. Notably, significant reductions in yield stress occurred between 5 and 9 min, between 9 and 11 min, and between 9 and 13 min of mixing. The pronounced decrease from 5 to 9 min implies substantial structural changes in the meat batter, likely due to protein denaturation and fat emulsification processes that reduce internal resistance to flow.

The decrease in yield stress with prolonged mixing time can be attributed to the mechanical disruption of muscle fibers and the formation of a more uniform meat batter. The slight increase in yield stress observed after 11 min may indicate the onset of overmixing, where excessive shear leads to protein network reformation, marginally enhancing the batter’s resistance to deformation [39]. The comparable rheological behavior between the control and experimental samples suggests that oleogel effectively mimics the functional properties of pork fat in the meat patties.

### 3.4. Microstructure of Control and Experimental Meat Patty Samples

**Figure 6:**
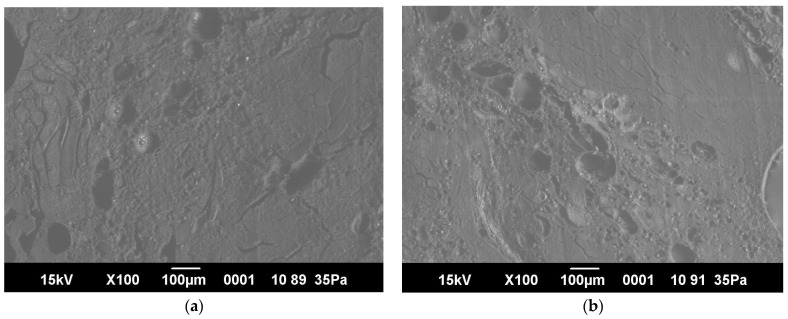

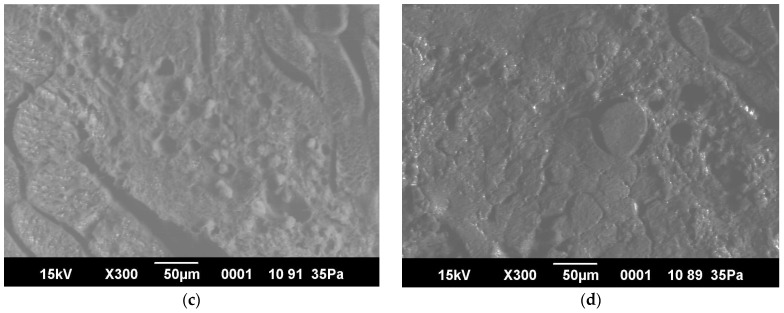
Microstructure images of meat patty cross-sections: (**a**) control sample (100× magnification), (**b**) experimental sample (100× magnification), (**c**) control sample (300× magnification), (**d**) experimental sample (300× magnification).

Figure 6 presents the microstructure images of the meat patty samples. The control sample exhibits noticeable porosity and large granules, which aligns with findings from previous studies [40,41]. The experimental sample exhibited a microstructure that is not significantly different from the structure of the control sample.

### 3.5. Sensory Characteristics of Control and Experimental Meat Patty Samples

**Figure 7:**
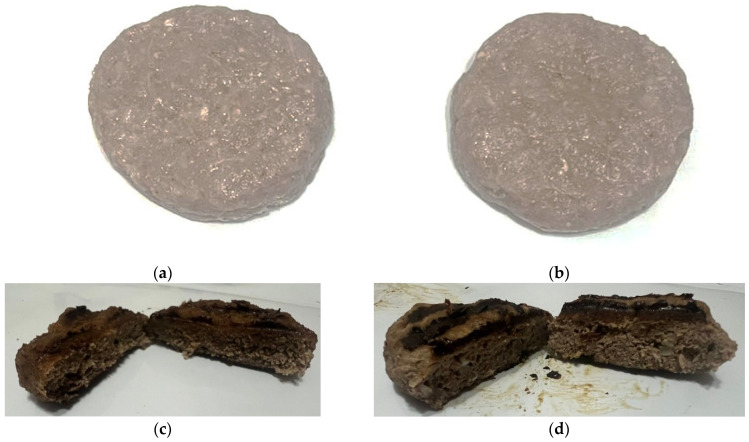
Heat treatment of meat patty samples: (**a**) control sample before treatment, (**b**) experimental sample before treatment, (**c**) control sample after treatment, (**d**) experimental sample after treatment.

Figure 7 illustrates the appearance of the patties before and after heat treatment, while the sensory characteristics of the control and experimental meat patties with oleogel addition are presented in Table 4.

A non-parametric Mann–Whitney *U* test was performed on the data in Table 4 because of the non-normal distribution within the dataset [42,43]. This statistical approach was used to analyze the sensory characteristics of the control and experimental meat patties. The analysis revealed highly significant differences in the appearance and aroma of the patties, with *p*-values of 0.035 and 0.022, respectively. This indicates that participants rated the experimental sample more favorably in terms of appearance and aroma, suggesting that the modifications to the sample had a positive effect on these sensory attributes.

The consistency of the meat patties also demonstrated a significant difference, with a *p*-value of 0.045. This finding supports the notion that the texture of the experimental patties was preferred by the participants, implying that the reformulated product could offer an improved overall eating experience.

Conversely, the color attribute yielded a *p*-value of 0.086, which did not reach statistical significance, suggesting that participants did not perceive a meaningful difference in color between the two samples. Similarly, taste did not show a significant difference, with a *p*-value of 1, indicating that both kinds of samples were rated equally in terms of flavor.

These results collectively suggest that the experimental treatments enhanced several key sensory characteristics, particularly appearance, aroma, and texture, which may increase consumer acceptance and market potential. The absence of significant differences in color and taste further suggests that these modifications did not detract from the overall flavor profile, maintaining consumer acceptability.

Regarding the yield stress data in Table 4, both the control and experimental samples displayed similar values during the initial mixing phase, indicating that the incorporation of oleogel does not significantly alter the immediate rheological properties of the meat batter. This finding is crucial for food manufacturers, as it demonstrates that oleogel can effectively replace pork fat without hindering the mixing process.

As mixing time increased, a gradual decrease in yield stress was observed, with the most pronounced decline occurring between 5 and 9 min of mixing. This reduction likely reflects significant structural transformations in the meat batter, such as protein denaturation and fat emulsification, leading to decreased internal resistance. This decrease in yield stress is beneficial, as it suggests that the mixing process is achieving its goal of fiber breakdown and improved homogeneity, resulting in better product texture.

However, the slight increase in yield stress observed after 11 min of mixing may indicate the onset of overmixing, where excessive shear forces start to reform the protein network. This highlights the importance of optimizing mixing times to avoid compromising texture. Understanding this phenomenon can help manufacturers strike a balance between achieving thorough mixing and preventing textural degradation, thereby enhancing product quality.

By identifying significant differences through the Mann–Whitney *U* test, food technologists and researchers can quantify the impact of oleogel substitution on meat patty characteristics. It is worth noting that *p*-values below 0.05 indicate statistically significant differences, which provides evidence that oleogel can positively influence the mechanical properties of the meat matrix and can therefore be used effectively as a substitute for conventional fat. These insights are valuable for product development, helping to establish optimal mixing conditions and ingredient interactions that yield desirable textural attributes in meat products.

Sensory evaluation plays a crucial role in assessing the acceptability of meat and derived products, focusing on characteristics such as color, texture, tenderness, juiciness, taste, and aroma [44].

The results of the organoleptic analysis indicate that the differences in aroma, color, and taste between the control sample and the experimental first-grade beef meat patties with 10% oleogel were minimal. On a five-point rating scale, the experimental sample scored higher in nearly all evaluated aspects, as Table 4 suggests. These findings are consistent with those reported in similar studies that utilized various oleogels [45,46].

The observed changes in the consistency and texture of the first-grade beef patties may be due to the interactions between the oleogel and meat proteins, which likely contribute to a firmer protein matrix in the final product [47]. The indicators obtained from the research meet the required standards and were validated through repeated trials.

### 3.6. Fatty Acids Composition of Meat Patties

The total amount of PUFAs in the experimental sample increased from 12.458% to 18.94% when pork fat was replaced with 10% oleogel. Notably, the amount of linoleic fatty acid, which is beneficial for human health, rose from 11.803% to 18.859%. In contrast, the content of SFAs decreased: stearic acid dropped from 15.675% to 4.124%, myristic acid declined from 1.529% to 0.87%, and margaric acid content was reduced from 0.335% to 0.091% (Table 5).

An analysis of the fatty acid composition revealed that the palmitic acid (C16:0) content increased in the experimental meat patties compared with the control samples. Specifically, the palmitic acid concentration was 27.98 g/100 g in the control patties and increased to 39.15 g/100 g in the experimental patties. The observed increase in palmitic acid in the experimental patties can be attributed to the fatty acid profile of the oleogel used as a fat replacer. Oleogels are structured oils that can mimic the functional properties of solid fats, and their fatty acid composition depends on the type of oil used in their formulation [48]. Despite the increase in palmitic acid, it is essential to consider the overall fatty acid profile and the balance between saturated and unsaturated fatty acids in the meat patties. 

In recent years, oleogel and fat emulsions have been added to food products. Such fats reduce the content of SFAs in food products and increase their functional properties. Solid fats obtained from vegetable oils by hydrogenation, transesterification, and fractionation processes are widely used for these purposes in various food products. In recent years, consumer awareness of the link between diet and health has increased, which may raise concerns about solid fats, including products high in SFAs. High LDL cholesterol levels are associated with an increased risk of cardiovascular diseases, including heart attacks and strokes [49,50,51].

Therefore, the complete replacement of pork fat with oleaginous fat in meat patties resulted in a product that is not only well-accepted but also has an increased content of unsaturated fatty acids.

The results of the Mann–Whitney *U* test conducted on the fatty acid composition of the meat patties revealed significant differences between the control and experimental samples. The concentrations of various fatty acids were evaluated, with *p*-values calculated to determine statistical significance. For SFAs, notable differences were observed: palmitic acid exhibited a highly significant change with a *p*-value of 0.024, and margaric acid similarly showed a significant difference with a *p*-value of 0.027, both indicating higher concentrations in the experimental group. Additionally, stearic acid presented an extremely low *p*-value of 0.0004, implying a strong statistically significant difference, where its concentration in the experimental sample was lower compared with the control sample.

In terms of MUFAs, palmitoleic acid showed a marked difference, with a *p*-value of 0.002, indicating a substantial variation between the two groups. However, when considering all MUFAs together, the overall *p*-value was 0.093, suggesting that changes in total monounsaturated content were not significant by conventional standards. For PUFAs, the overall difference was significant, with a *p*-value of 0.024. This category included linoleic acid, which had a *p*-value of 0.048, demonstrating a significant difference in concentration between the experimental and control samples. Additionally, γ-linolenic acid showed significance at a *p*-value of 0.014.

Table 5 provides crucial insights into the nutritional profile of the meat patties, particularly in light of consumer interest in health-conscious dietary options. The data illustrate how replacing pork fat with oleogel influences fatty acid composition, notably increasing unsaturated fatty acids while decreasing saturated ones. This substitution is designed to enhance the health benefits of the product.

A critical observation from the data is the substantial increase in PUFAs, rising from 12.458% in the control sample to 18.94% in the experimental sample. This increase is significant, as PUFAs—especially linoleic acid—are known for their cardiovascular benefits and anti-inflammatory properties. The increase in linoleic acid from 11.803% to 18.859% underscores the potential of the experimental patties to contribute positively to consumer health, addressing concerns about the intake of saturated fats and their association with elevated LDL cholesterol levels.

The table also highlights a significant reduction in total SFAs, which aligns with current dietary guidelines emphasizing reduced saturated fat consumption. For example, stearic acid decreased significantly from 15.675% to 4.124%, and myristic acid declined from 1.529% to 0.87%. These reductions suggest that the experimental patties adhere more closely to recommended dietary patterns, which positions them as a healthier option in the market. The ability to lower saturated fat content while preserving product quality is a valuable competitive advantage in the food industry.

#### Statistical Implications

The results of the Mann–Whitney *U* test underscore the efficacy of oleogel substitution in achieving desirable health outcomes. The significant differences observed confirm that the alterations in fatty acid concentrations are statistically meaningful. This evidence provides a solid basis for food scientists and product developers to promote the experimental patties as a healthier alternative, backed by empirical data on their improved fatty acid profile.

Moreover, Table 5 offers valuable insights for product formulation strategies aimed at enhancing consumer acceptance and satisfaction. Understanding the specific shifts in fatty acid composition can guide further research into optimizing oleogel formulations. By selecting appropriate oil sources, manufacturers can maximize health benefits while maintaining desirable sensory qualities. The increase in PUFAs can also be leveraged in marketing campaigns to highlight the enhanced nutritional value of the experimental product compared with traditional meat patties.

## 4. Conclusions

The findings from the study demonstrate that incorporating 10% oleogel into first-grade beef meat patties effectively enhances key quality attributes. Specifically, the inclusion of oleogel improves moisture-binding capacity, contributes to a more desirable texture and consistency, and significantly enriches the patties with beneficial PUFAs. Importantly, the substitution of pork fat with oleogel did not lead to significant alterations in fundamental physicochemical and structural properties, such as pH and moisture-binding capacity. This suggests that the basic integrity of the meat product is preserved, even with this healthier fat replacement.

The foregoing analysis revealed a noteworthy reduction in several SFAs, including capric, lauric, myristic, margaric, stearic, heneicosanoic, and behenic acids. Concurrently, there was a marked increase in PUFAs, particularly linoleic and arachidonic acids, which aligns with current dietary guidelines that emphasize the reduction of saturated fat intake and promotion of healthier unsaturated fats. This shift in fatty acid composition underscores the nutritional advantage of using oleogel, presenting a viable strategy for developing lower-fat meat products that meet consumer health expectations.

From a practical standpoint, the ability to improve the health profile of meat patties without compromising essential quality characteristics offers significant market potential. These oleogel-enhanced patties could attract health-conscious consumers, which renders them a valuable addition to the portfolio of meat products designed for improved nutritional value.

To build on these promising outcomes, future investigations could explore optimizing the oleogel formulation to maximize protein retention and further enhance sensory properties, such as flavor and juiciness. Additionally, it would be beneficial to examine the long-term storage stability and consumer acceptability of these modified patties in real-world settings. Assessing the impact of varying oleogel concentrations and types of oils used in the oleogel matrix could also provide deeper insights into creating even more effective and appealing fat substitutes.

## 5. Patents

Igenbayev Aidyn Kairbekovich, Ospankulova Gulnazym Khamitovna, Temirova Indira Zhanatovna, Aldieeva Akmaral Beyimbetovna, Kozhamsugirov Kerimbek Musayevich, Amirkhanov Shyngys Amirzhanuly, Shaymerdenov Zhumabek Nauryzbayevich. “Method of Production of Semi-Smoked Sausages with Low Saturated Fat Content”. Patent application filed on 24 September 2023.

## Figures and Tables

**Table 1:** Meat patty composition.

Ingredient	Control Sample	Experimental Sample
**Main Ingredients (%)**		
First-Grade Beef	60	60
Pork	30	30
Pork Fat	10	—0
Oleogel	0	10
**Additional Ingredients (g)**		
Salt	1.8	1.8
Onion	2	2
Black Pepper	0.5	0.5
Garlic	0.3	0.3

**Table 2:** Physicochemical characteristics of beef meat patties.

Meat Sample	Moisture (%)	Protein (%)	Fat (%)	Carbohydrate (%)	Ash (%)	pH
Control	56.42 ± 0.95 ^a^	20.16 ± 0.33 ^b^	19.95 ± 0.34 ^b^	1.65 ± 0.05 ^a^	1.82 ± 0.07 ^a^	6.27 ± 0.12 ^b^
Experimental	57.62 ± 0.86 ^b^	19.47 ± 0.28 ^a^	18.37 ± 0.26 ^a^	2.56 ± 0.09 ^b^	1.98 ± 0.06 ^a^	6.02 ± 0.17 ^a^

Note: Different superscript letters (a,b) within the same column indicate statistically significant differences between the control and experimental samples (*p* < 0.05). All results are given in the form mean value ± standard deviation.

**Table 3:** Yield stress of the meat batter.

Mixing Time (min)	Yield Stress (Pa)	
	Control	Experimental
5	604 ± 7 ^a^	587 ± 6 ^a^
7	575 ± 8 ^a^	562 ± 6 ^a^
9	401 ± 7 ^b^	382 ± 4 ^b^
11	369 ± 6 ^c^	327 ± 6 ^c^
13	386 ± 6 ^c^	351 ± 5 ^d^

Different superscript letters (a–d) within the same column indicate statistically significant differences. (*p* < 0.05). All results are given in the form mean value ± standard deviation.

**Table 4:** Sensory characteristics of the control and experimental meat patty samples.

Meat Sample	Control	Experimental
Appearance	4.7 ± 0.11 *	4.8 ± 0.08
Color	4.9 ± 0.07	5 ± 0.09
Taste	5 ± 0.09	5 ± 0.06
Aroma	4.7 ± 0.09	4.9 ± 0.06
Consistency	4.9 ± 0.06	5.0 ± 0.09

* Mean values are the scores given by participants in the sensory group on a five-point scale; *n* = 21 for each type of assessment. All results are given in the form mean value ± standard error.

**Table 5:** Fatty acid composition of the meat patties.

Fatty Acid	Control Sample	Experimental Sample
Fatty Acid Composition (g/100 g)		
Saturated Fatty Acids	46.197 ± 2.31	44.371 ± 2.219
C 10:0 Capric	0.062 ± 0.003 ^a^	nd *
C 12:0 Lauric	0.071 ± 0.004 ^b^	nd
C 14:0 Myristic	1.529 ± 0.076	0.87 ± 0.044
C 15:0 Pentadecylic	0.043 ± 0.002	nd
C 16:0 Palmitic	27.987 ± 1.399	39.157 ± 1.958
C 17:0 Margaric	0.335 ± 0.017	0.091 ± 0.005
C 18:0 Stearic	15.675 ± 0.784	4.124 ± 0.206
C 20:0 Arachidic	nd	0.063 ± 0.003
C 21:0 Heneicosanoic	0.457 ± 0.023	nd
C 22:0 Behenic	0.038 ± 0.002	nd
C 23:0 Tricosanoic	nd	0.07 ± 0.004
**Monounsaturated Fatty Acids**	**41.344 ± 2.06** **6**	**36.689 ± 1.834**
C 16:1 (Cis-9) Palmitoleic	1.764 ± 0.088	0.135 ± 0.007
C 17:1 (Cis-10) Heptadecenoic	0.287 ± 0.014	nd
C 18:1 (Cis-9) Oleic	38.64 ± 1.932	36.477 ± 1.824
C 20:1 (Cis-11) Eicosenoic	0.606 ± 0.03	0.077 ± 0.004
C 22:1 (Cis-13) Erucic	0.047 ± 0.002	nd
**Polyunsaturated Fatty Acids**	**12.45** **9 ± 0.6** **8**	**18.94** **0 ± 0.947**
C 18:2 n-6 t Trans-Linoleic	0.043 ± 0.003	nd
C 18:2 n-6 c Linoleic	7.519 ± 0.59	11.611 ± 0.943
C 18:3 n-6 γ-Linolenic	0.554 ± 0.028	0.081 ± 0.004
C 20:3 n-6 c (Cis-8,11,14) Eicosatrienoic	0.059 ± 0.003	nd
C20:4 n-6 Arachidonic	4.284 ± 0.004	7.248 ± 0.012

* nd—not detected. Note: Different superscript letters (a–b) within the same column indicate a statistically significant difference.

## Data Availability

The original contributions presented in this study are included in the article. Further inquiries can be directed to the corresponding author.
